# Learning deliberate reflection in medical diagnosis: does learning-by-teaching help?

**DOI:** 10.1007/s10459-022-10138-2

**Published:** 2022-08-01

**Authors:** Josepha Kuhn, Silvia Mamede, Pieter van den Berg, Laura Zwaan, Petra van Peet, Patrick Bindels, Tamara van Gog

**Affiliations:** 1 Department of General Practice, Erasmus Medical Centre, Rotterdam, The Netherlands; 2 Institute of Medical Education Research Rotterdam, Erasmus Medical Centre, Rotterdam, The Netherlands; 3 Department of Education, Utrecht University, Utrecht, The Netherlands; 4 Department of Public Health and Primary Care, Leiden University Medical Centre, Leiden, The Netherlands

**Keywords:** Reflective reasoning, Critical thinking, General practice, Diagnostic error, Instructional design

## Abstract

Deliberate reflection has been found to foster diagnostic accuracy on complex cases or under circumstances that tend to induce cognitive bias. However, it is unclear whether the procedure can also be learned and thereby autonomously applied when diagnosing future cases without instructions to reflect. We investigated whether general practice residents would learn the deliberate reflection procedure through ‘learning-by-teaching’ and apply it to diagnose new cases. The study was a two-phase experiment. In the learning phase, 56 general-practice residents were randomly assigned to one of two conditions. They either (1) studied examples of deliberate reflection and then explained the procedure to a fictitious peer on video; or (2) solved cases without reflection (control). In the test phase, one to three weeks later, all participants diagnosed new cases while thinking aloud. The analysis of the test phase showed no significant differences between the conditions on any of the outcome measures (diagnostic accuracy, *p* = .263; time to diagnose, *p* = .598; mental effort ratings, *p* = .544; confidence ratings, *p* = .710; proportion of contradiction units (i.e. measure of deliberate reflection), *p* = .544). In contrast to findings on learning-by-teaching from other domains, teaching deliberate reflection to a fictitious peer, did not increase reflective reasoning when diagnosing future cases. Potential explanations that future research might address are that either residents in the experimental condition did not apply the learned deliberate reflection procedure in the test phase, or residents in the control condition also engaged in reflection.

**Supplementary Information:**

The online version contains supplementary material available at 10.1007/s10459-022-10138-2.

## Introduction

Reflection upon one’s own experiences has been much valued as a means for physicians to learn and improve performance throughout their professional life (Mann et al., 2009; Ng et al., 2015). Reflection may have different foci, occur at different moments of practice, and there are many ways for physicians to engage in reflection (Ng et al., 2015). While reflection in a broader sense can be seen as the ability to critically examine one’s own explanation for or beliefs about a problem (Dewey, 1910), the *deliberate reflection* (Mamede et al., 2008a) procedure has been developed to facilitate structured reflection and avoid biased reasoning on to-be-diagnosed clinical cases. In two recent reviews about the effectiveness of cognitive interventions to improve diagnostic accuracy, this procedure showed to be among the most effective and consistently successful interventions to improve diagnostic accuracy (Lambe et al., 2016; Prakash et al., 2019). Deliberate reflection consists of specific instructions for stepwise consideration of initial diagnostic hypothesis and alternative diagnoses. Physicians first read the case and give an initial diagnosis. After that, they are asked to list all the findings that speak for and against their initial diagnosis for the case, as well as findings that they would expect with their diagnosis, which are absent. Then they are asked to generate alternative diagnoses and do the same ‘reflective steps’ for those. When a couple of diagnoses have been analysed, they rank the diagnoses in order of likelihood to make a decision on their final diagnosis. This procedure aims to stimulate physicians to reflect on their first impression of a case to avoid excessive reliance on intuitive reasoning.

Deliberate reflection has been shown to improve diagnostic accuracy, especially when cases are complex (Mamede et al., 2008a, 2010a), or when physicians diagnose cases under conditions that tend to induce cognitive biases that mislead diagnostic reasoning (Mamede et al., 2010b; Schmidt et al., 2014, 2017). For example, when they have just recently seen a case that resembles the one at hand on superficial features, deliberate reflection helps physicians not to be misled by these similarities into thinking that they have the same clinical condition when they do not, or when patients show disruptive behaviour, deliberate reflection can help physicians to better focus on the clinical findings and avoid diagnostic error. These studies have mainly focussed on a direct improvement in performance (i.e., diagnostic accuracy on the case reflected upon). However, it is as yet unclear whether the procedure itself can be learned and would then be applied autonomously (i.e., without reflection instructions) when diagnosing future cases. It has been questioned whether reasoning processes can be taught at all, as physicians engage in it unconsciously and interventions to teach diagnostic reasoning have often been found to be ineffective in improving diagnostic accuracy (Norman et al., 2017; Schmidt & Mamede, 2015). On the other hand, literature on example-based learning shows that specific procedures can be learned and applied to new problems, and that this does not only apply to cognitive skills, for example in physics (Hoogerheide et al., 2019a) or mathematics (Paas, 1992), but also to higher order skills such as collaboration (Rummel & Spada, 2005). Therefore, similar interventions may be useful to teach the steps of the deliberate-reflection procedure.

Example-based learning has proven very effective and efficient for many types of cognitive and higher order skills (Atkinson et al., 2000). However, in a previous study, the attempt to teach deliberate reflection by studying examples of experts’ reflection on cases proved to be ineffective (Kuhn et al., 2020). Perhaps studying the examples was not sufficiently challenging. In order to learn a new problem-solving procedure and be able to transfer it to novel problems, students should actively engage with the study material. Learning-by-teaching could improve the effectiveness of example-based learning as it stimulates such active engagement. The present study investigated whether learning-by-teaching (Fiorella & Mayer, 2013, 2014; Hoogerheide et al., 2014, 2016), an instructional method that has proven effective for enhancing learning and transfer to novel contexts, would be an effective way to learn (to adopt) the procedure.

Previous studies have found, that when students study material with the expectation to teach, this alone can have a short-term benefit on learning (Brown & Kane, 1988; Fiorella & Mayer, 2013). When students then also teach the material, they seem to develop a deeper understanding of the material and a benefit on learning is found even after a one-week delay. Explaining study material helps students to actively process it and to understand its important aspects and underlying rationale (Fiorella & Mayer, 2014). This helps with learning of a new problem-solving procedure and with applying it to slightly novel problems (i.e., transfer). Another benefit of learning-by-teaching is that students practice to retrieve the material from memory while teaching (Van Gog & Rummel, 2010) which improves long term retention of the material (see testing effect; Koh et al., 2018). Some studies have included measures of perceived mental effort because in combination with performance it can help to investigate the efficiency of the instructional method (Roediger & Karpicke, 2006). These studies found, that learning-by-teaching is typically more cognitively demanding than restudying the material (i.e., participants usually perceive teaching as being more effortful), but this additional effort pays off, as they show better learning results (Hoogerheide et al., 2016, 2019a; Van Gog & Paas, 2008). Furthermore, it has been found to be more effective if students have a (perceived) audience (in the form of a camera), than when they teach without audience. The reason for this may be, that this feeling of a *social presence* of an audience increases active processing of the material (Hoogerheide et al., 2019b) and arousal (Hoogerheide et al., 2016), which can foster learning.

We build on a recent study (Hoogerheide et al., 2019a, 2019b), in which psychology students were taught how to solve physics problems through learning-by-teaching: they first studied an example and then recorded a video on which they explained to a fictitious peer how to solve the problem. On a post-test, students who engaged in learning-by-teaching outperformed students who studied an additional example instead.

In the present study, we investigated whether general practice residents, i.e. physicians in training to become specialists, would learn the method of deliberate reflection by studying three clinical cases presented as examples of deliberate reflection and subsequently explaining the procedure on video (compared to a control group that only diagnosed clinical cases). On a post-test one to three weeks later, all participants diagnosed a new set of (test) cases while thinking aloud (to capture the residents’ reasoning process; Durning et al., 2013). We hypothesized that participants in the learning-by-teaching condition would have learned and would apply deliberate reflection on the test cases, meaning they would engage in more reflective reasoning when diagnosing cases in the test phase (as indicated by the think-aloud protocols) and, therefore, would take more time to diagnose and show higher diagnostic accuracy than participants in the control condition. For additional measures on the learning process and outcome, we measured mental effort (Van Gog & Paas, 2008), an indicator of experienced cognitive load, and confidence in the given diagnosis.

## Method

### Participants and design (Fig. 1)

**Fig. 1:**
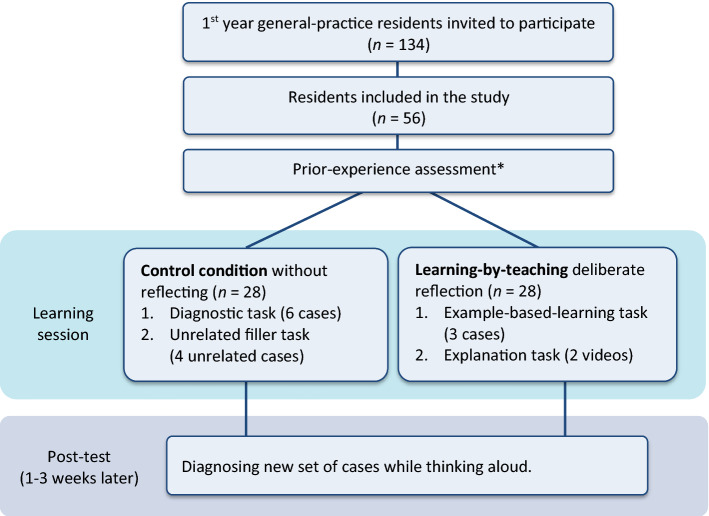
Illustration of the study protocol. The prior-experience questionnaire was only filled in by 35 of the 56 participants.

Ninety-nine residents followed our invitation and came to the first study session, and 56 of them (39 female; age *M* = 29.05, *SD* = 2.85) completed both sessions. The residents were in the first year of a three-year residency program at either the Erasmus Medical Centre in Rotterdam (*n* = 37), or the Leiden University Medical Centre (*n* = 19). The study took place during the usual educational program and participants did not receive compensation. The ethics committee of the Department of Psychology, Erasmus University Rotterdam, approved the study. Participants were randomly assigned to the learning-by-teaching condition (*n* = 28) or the control condition (*n* = 28).

### Materials

*Prior knowledge questionnaire* To check if there were no differences in prior knowledge between the experimental and control condition, participants filled out a prior knowledge questionnaire. Besides demographics and experience in clinical practice, participants were presented with a list of clinical symptoms and conditions (Supplementary material, Table 1) including those presented in the cases of this study and others (i.e. fillers) to disguise the diseases of interest. The participants were asked to indicate their experience on a 5-point Likert-scale ranging from 1 (I have never seen a patient with this condition, symptom, or complaint) to 5 (I have seen many patients with this condition, symptom, or complaint).

*Cases* In this study we used ten written, clinical cases (Supplementary material, Table 2) which described complex problems as they can be encountered in general practice (example in supplementary material, Fig. 1). Each case described a different patient with complaints, medical history, and findings from physical examination, and in some cases additional test results. The cases were prepared by experienced general practitioners. For validation, each case was solved by two different general practitioners who were blind to the intended diagnosis. If one or both general practitioners did not give the intended diagnosis, they discussed and adjusted the case until they reached agreement.

*Deliberate Reflection examples* For the experimental condition, we used a combination of ‘example-based learning’ (Van Gog et al., 2019) and ‘learning-by-teaching’ (Fiorella & Mayer, 2013, 2014; Hoogerheide et al., 2014, 2016), so participants could first study examples of the procedure that they would be asked to teach. For this, three written worked-out examples were used, which illustrated how deliberate reflection was applied on a case of the learning phase (example in supplementary material, Fig. 2). Each example showed the reflection procedure on a different case from the learning phase and analysed three plausible differential diagnoses. The deliberate reflection method aims at inducing a critical review of the initial and following diagnoses (Mamede et al., 2008a). The procedure requires the physician to first read a case and give an initial diagnosis. Subsequently, the physician goes back to the case and lists (1) findings that support the diagnosis, (2) findings that oppose the diagnosis, (3) findings that would have been expected if the diagnosis were true, but were absent. The physician then gives (4) an alternative diagnosis and follows the same analytical steps (1–4) for this alternative diagnosis, and for a third diagnosis. The written deliberate reflection examples left out the final step of deliberate reflection, which is the ranking of the diagnoses in order of likelihood and thereby choosing a final diagnosis, as the residents were asked to do this themselves. The three worked-out examples were prepared by experienced general practitioners.

*Mental effort and confidence* To acquire additional information on the reasoning process, participants were asked to rate their mental effort when diagnosing as well as their confidence in their final diagnosis. Mental effort and confidence were each rated on different pages and on 9-point-Likert-scales ranging from 1 (very low) to 9 (very high), similar to the mental-effort rating by Paas (1992).

*Explanation videos* Using a web cam recorder (www.addpipe.com), participants in the learning-by-teaching condition were instructed to record two videos in the learning phase, addressing a fictitious peer. For the first video, they were shown an empty reflection table, which had the same format as the deliberate reflection examples, but all text was removed. Participants were asked to explain what the steps of deliberate reflection are and how the procedure can help to avoid common reasoning errors. For recording the second video, they were shown one of the cases they had diagnosed earlier, together with a table containing only the steps of deliberate reflection. Participants now had to explain how the given case was diagnosed by applying the deliberate reflection procedure.

*Presentation* The prior-knowledge questionnaire and the two study sessions were programmed in Qualtrics software (Version 05.2018). Two versions of each session presented the cases of a session in a different order to reduce the influence of item-order effects on participants’ answers. During the learning phase, participants in the learning-by-teaching condition saw three cases together with the worked-out reflection examples for these cases. Participants in the control condition saw the same three cases without reflection example, and three additional cases. Each session was self-paced and participants could not move back in the program. The participants’ answers and response times were saved automatically.

## Procedure

Prior to the study, participants had been told that we investigated diagnostic reasoning and the effectiveness of educational methods. Approximately two weeks before the first experimental session, the residents received a Qualtrics questionnaire per email, and were asked to fill it in prior to the session. The experimental sessions were conducted at the residents’ institute and were led by different researchers all following the same instructions. At the beginning of the first session, participants were randomly distributed. Residents in the experimental group received instructions for the learning-by-teaching condition and residents in the other group for the control condition. First, all residents individually watched an instruction video on the computer which explained how an example case had been diagnosed following the instructions of their study condition. In the learning-by-teaching condition, the video therefore explained the steps of deliberate reflection and how they could help to avoid common reasoning errors. After watching the video, all participants started with the diagnostic task.

In the control condition, participants were shown the first case. They were asked to read the case until they had decided which diagnosis is the most likely for the case and then to move on to the next page. The case disappeared from the screen and they were asked to fill in the diagnosis. On the following two pages they were asked to rate how much mental effort they invested in diagnosing the case and how much confidence they had in the diagnosis. After this, they moved on to the next case until all six cases had been diagnosed. Participants in the control condition analysed three cases more than participants in the learning-by-teaching condition. These cases had the same structure but a different content (Supplementary material, Table 2). As a second measure to keep the time-on-task the same for both conditions, participants in the control group then did a filler task, in which they diagnosed four unrelated internal medicine cases. These cases described patients with acute prostatitis, acute glomerulonephritis, hepatitis B and deep vein thrombosis.

Participants in the learning-by-teaching condition, were shown the first case and were asked to read it and give a diagnosis (as in the control condition). They then saw the case again along with a worked-out deliberate reflection example. Participants were asked to study this example and to rank the given diagnoses in order of likelihood. Then, they rated their mental effort and confidence, and went on to the next case until all three cases were diagnosed. When finished, participants moved on to a task wherein they recorded the two explanation videos, addressing a fictitious peer.

One to three weeks later, the test session took place (the timing difference was due to differences in the residents’ class schedule). The test was the same for both conditions. Participants were asked to diagnose new cases while thinking aloud. In order to get used to the method, they did two unrelated think-aloud tasks without clinical cases. After this, they started to diagnose four new cases. Participants started the audio recorder and then saw a case. They were asked to think aloud until they had arrived at their final (most likely) diagnosis for the case. They went on to the next page and filled in this diagnosis. After this, they rated their mental effort and confidence and went on to the next case until all four cases had been diagnosed. Finally, participants received a written debriefing and were thanked for their participation.

## Data analysis

For all analyses, we used a significance level of *α* = 0.05 and did a Bonferroni correction for the number of tests, which led to α = 0.001. As a measure of effect size, *ηp*^*2*^ is provided for the analyses of variances, with 0.01, 0.06, 0.14 corresponding to small, medium and large effects (Cohen, 1988).

*Prior knowledge* Mean prior experience ratings were computed for the chief complaints and diagnoses of the cases. To check for initial differences between the groups, we conducted a one-way analysis of variance (ANOVA) on the mean prior experience ratings with condition (learning-by-teaching, control) as a between-subjects factor.

*Learning phase* To check whether participants had learned the deliberate reflection procedure and whether they completed the explanation task appropriately, we analysed the first explanation video recorded under the learning-by-teaching condition, wherein residents had to explain the steps of deliberate reflection. Due to technical problems only 17 videos were recorded correctly and could be used for analysis. Two researchers independently judged whether residents named the four steps of deliberate reflection and in which order to use them. The two raters completely agreed when scoring the deliberate reflection steps and had an almost perfect interrater reliability (Landis & Koch, 1977) for scoring whether the correct order was given, *Kappa* = 0.87.

*Test phase* Participants’ final diagnoses were scored by two general practitioners independently as either 1 (correct core diagnosis), 0.5 (partially correct), or 0 (incorrect). The interrater reliability was excellent, *ICC* = 0.94 (Cicchetti, 1994), and disagreements were later resolved through discussion. Furthermore, we analysed how much time participants had spent on a case until they moved to the next page to fill in a diagnosis (time to diagnose). Participants’ mean scores on the test cases were computed on diagnostic accuracy, time to diagnose, mental effort, and confidence. To analyse differences between the two conditions, we conducted a one-way ANOVA on each outcome measure.

Further, we analysed the recordings from the think-aloud task, to test whether the deliberate reflection procedure was adopted when diagnosing test cases one to three weeks later. We were missing 66 recordings (29%) due to technical errors. The remaining 158 recording from 46 participants first were transcribed. We then counted the numbers of idea units (Meyer, 1975; Schiefele & Krapp, 1996) in the think-aloud protocols. An idea unit is the smallest meaningful idea that can be identified in a fragment of text. The idea units were coded according to the deliberate reflection steps 1–4. Thereby, a table as shown as deliberate reflection example was reconstructed from the residents’ think-aloud protocols. Consequently, an idea unit that was categorised as step 1–3 could be counted more often than it was vocalised. That was the case when a resident linked one argument to multiple diagnoses. Two researchers who were blind to the condition categorised and counted the idea units without judging the correctness of the medical content. A sample of 10% of the data was rated by both researchers with an interrater reliability ranging from fair to excellent (Cicchetti, 1994), step 1 to 4: *ICC* = 0.60, *ICC* = 0.84, *ICC* = 0.43, *ICC* = 0.68.

From these idea units, we computed a measure that reflects how many key elements of deliberate reflection were used when solving a case. A crucial element is, that participants do not only consider information that supports their diagnosis at hand, but that they consider contradictory arguments and alternative diagnoses, as well, to critically reflect on their diagnosis. The aim of these steps is to help physicians to avoid a tunnel vision and confirmatory bias towards their first impression of the case, as these types of reasoning flaws have been associated with diagnostic errors (Hoogerheide et al., 2019a). Therefore, we analysed the number of *contradiction units* in the participants’ reasoning to measure adoption of the deliberate reflection procedure. Contradiction units were defined as the idea units that we categorised into the deliberate reflection step 2 (what speaks against), 3 (what is missing), and 4 (differential diagnoses). For the statistical analysis, the *proportion of contradiction units* was calculated relative to all idea units given by the participant (this adjusts for possible differences between cases in the total number of idea units reported). From this, we computed the participants’ mean proportion of contradiction units. A one-way ANOVA was conducted on mean proportion of contradiction units with condition (learning-by-teaching, control) as a between-subjects factor.

## Results

### Prior clinical experience

Table﻿ ﻿1 shows the descriptive statistics and experience with the medical conditions and complaints. Note that only 35 of the 56 participants filled in the prior knowledge assessment. The conditions did not significantly differ on prior experience with the symptoms, *F* (1, 33) = 2.01, *p* = 0.150, *η*_*p*_^*2*^ = 0.06, or with the diagnoses *F* (1, 33) = 0.01, *p* = 0.922, *η*_*p*_^*2*^ < 0.01.

**Table 1:** Prior experience rating of the symptoms and correct diagnoses presented in this study

	N	All cases	
		Mean	SD
*Age*			
Control	28 (17 female)	30.21	3.23
Learning-by-teaching	28 (22 female)	27.78	1.66
Total	56 (39 female)	29.05	2.85
*Prior experience with symptoms*			
Control	20	3.05	.43
Learning-by-teaching	15	3.25	.39
Total	35	3.14	.42
*Prior experience with diagnoses*			
Control	20	2.57	.59
Learning-by-teaching	15	2.55	.51
Total	35	2.56	.55

Participants indicated their experience on a 5-point Likert-scale ranging from 1 (I have never seen a patient with this condition, symptom, or complaint) to 5 (I have seen many patients with this condition, symptom, or complaint)

### Learning phase

Out of the 17 explanation videos we analysed, 11 residents described the procedure perfectly. Four residents described all steps but did not state clearly that you should first analyse one diagnosis and only after that think of the next diagnosis. This might be important for deliberate reflection to be effective (Mamede & Schmidt, 2014). Two residents did not state clearly that when falsifying one’s diagnosis you should include symptoms that you would have expected if the diagnosis was true but were absent in the case.

### Test phase

Table 2 shows the mean and standard deviation of diagnostic accuracy, mental effort, confidence, time to diagnose, and proportion of contradiction units that were measured during the post-test. One-way ANOVAs showed no main effects of condition on diagnostic accuracy, *F* (1, 54) = 1.28, *p* = 0.263, *η*_*p*_^*2*^ = 0.02, on time to diagnose, *F* (1, 54) = 0.28, *p* = 0.598, *η*_*p*_^*2*^ < 0.00, on mental effort ratings, *F* (1, 54) = 0.37, *p* = 0.544, *η*_*p*_^*2*^ = 0.01, on confidence ratings, *F* (1, 54) = 0.14, *p* = 0.710, *η*_*p*_^*2*^ < 0.01, or on the proportion of contradiction units, *F* (1, 43) = 0.37, *p* = 0.544, *η*_*p*_^*2*^ = 0.01. Note that only 45 residents were included in the latter analysis because for 11 residents the think-aloud task was not recorded correctly.

**Table 2:** All outcome measures collected during the post-test

	N	All cases	
		Mean	SD
*Diagnostic accuracy*			
Control	28	.51	.30
Learning-by-teaching	28	.59	.23
Total	56	.55	.27
*Time to diagnose*			
Control	28	271.00	86.41
Learning-by-teaching	28	260.64	56.68
Total	56	265.82	72.59
*Mental effort*			
Control	28	5.40	1.37
Learning-by-teaching	28	5.59	1.01
Total	56	5.49	1.20
*Confidence*			
Control	28	5.62	1.00
Learning-by-teaching	28	5.72	1.14
Total	56	5.67	1.06
*Proportion of contradiction units*			
Control	22	.29	.10
Learning-by-teaching	24	.31	.09
Total	46	.30	.10

Diagnostic accuracy was scored as 0 (incorrect), 0.5 (partially correct), or 1 point (correct). Time to diagnose was measured in seconds. Mental Effort and Confidence were rated on a 9-point Likert-scale ranging from 1 (very low) to 9 (very high)

## Discussion

Although prior studies have shown that deliberate reflection improves diagnosis (Mamede et al., 2008a, 2010a, 2010b; Schmidt et al., 2014, 2017), it is as yet unclear whether the deliberate reflection procedure can be learned and autonomously applied on future cases (without prompting physicians to do so). We therefore investigated whether general practice residents would learn the deliberate reflection procedure by studying examples and explaining the procedure on video (compared to a control group that only diagnosed cases) and apply it when solving novel cases one to three weeks later. There were no differences between the learning-by-teaching condition and the control condition in the proportion of contradictory idea units reported while diagnosing the case, time needed to diagnose, and diagnostic accuracy. Practicing with deliberate reflection also did not influence participants’ confidence in their diagnosis or mental effort needed to solve future cases. Against our expectations, these findings suggest that the two conditions did not differ in the extent to which they incorporated elements of the deliberate reflection procedure in their reasoning process.

One possible explanation is that all the residents already naturally engage in reflective reasoning. The cases in this study were designed to be difficult and to be more complex than in clinical practice, because complexity is known to trigger reflective reasoning (Mamede et al., 2007, 2008b, 2010a). Moreover, the request to diagnose the cases while thinking aloud probably also induced a more thorough consideration of the case than what they would naturally do. Perhaps these cases stimulated reflection in all the residents. If the residents in the control condition reasoned similarly to those in the learning-by-teaching condition, who had learned which reasoning steps help them to prevent errors, this means that the residents could already engage in some sort of reflective reasoning. Therefore, deliberate reflection might not further improve the residents’ diagnostic reasoning. This explanation is supported by comments from the residents’ teachers who said that they always expect their trainees to generate multiple differential diagnoses for a case. Thus, it is possible that their education already implies the steps of deliberate reflection to some degree and that residents in this phase of postgraduate training are able to reflect and therefore engage in reflective reasoning when solving cases that are not straightforward.

An alternative explanation is that residents in the learning-by-teaching condition did learn the deliberate reflection procedure but did not apply it during the test phase. The videos of the first explanation task suggest that the residents had learned the deliberate reflection procedure. However, in order to adopt it as a diagnostic strategy for themselves, perhaps they would need more practice with the procedure (i.e., automatize it), with a shorter time interval between the sessions. Future studies could test whether a learning phase with multiple sessions would be effective for residents in adopting deliberate reflection. In contrast to prior studies (Graber et al., 2005; Hoogerheide et al., 2019a) the participants in this study did not have fixed times to study or explain the learned material. We do not know whether a fixed study period would have helped participants to make better use of their study opportunity. It may also be that the residents did not feel the need to engage in reflection. As we explained above, the cases were prepared to be difficult, because higher difficulty levels tend to trigger reflection (Mamede et al., 2007, 2008b) and the residents’ diagnostic accuracy showed to be at an intermediate level, at which deliberate reflection has been shown to be beneficial (Fiorella & Mayer, 2013). However, it is also known that physicians’ perception of how difficult a case is far from an objective, accurate judgement (Meyer et al., 2013). Perhaps the residents in our study did not perceive the cases as demanding enough to require further thinking.

Another explanation is that, though residents in the learning-by-teaching condition did learn the deliberate reflection procedure, this does not mean that they have learned to adopt the procedure as a general reasoning process for addressing future problems. While cognitive interventions can improve diagnostic accuracy when physicians are explicitly instructed to use them (Costa Filho et al., 2019; Lambe et al., 2016) it has been questioned whether generalizable cognitive skills that could be applied to new problems, can be taught (Eva et al., 1998; Monteiro et al., 2020; Prakash et al., 2019). Content specific interventions that increase or reorganize medical knowledge may be more effective to improve diagnostic accuracy (Norman, 1988; Schmidt & Mamede, 2015). Hoogerheide et al. (2019a, 2019b) may have found transfer of the learned problem-solving procedure to novel problems because their learning problems and test problems were more similar in content than the different cases in the present study were. A limitation of the study is the substantial drop-out from the first to the second session, which reduced our sample size. The missing think-aloud data, that could not be analysed, further reduced our sample size, which may have caused the study power to be insufficient to find an existing effect. Besides that, we do not know whether the think-aloud task in the test phase affected the residents’ reasoning and fostered reflective reasoning of all residents. Being required to think aloud while reasoning naturally leads to considering case findings more extensively, eventually ‘removing’ physicians from an intuitive reasoning mode. Furthermore, we do not know whether four cases in the test phase were enough to find a possible difference between the conditions. Another limitation is that we have no objective standard of what can be considered much or little reflection. As both conditions performed the same, we cannot say whether this is because both engaged in much or little reflective reasoning. Future studies should include a reflection template to which the participants’ reasoning can be compared. Furthermore, qualitative studies could give more insight into the reasoning process.

Given that our residents might perhaps already have had too much experience with reflection, it would be interesting for future research to test whether learning-by-teaching, which seems to be particularly effective for students with little prior knowledge (Hoogerheide et al., 2019a, 2019b), would be effective to teach deliberate reflection to medical students. Ibiapina et al. (2014) conducted a study among students in which they focused on effects of deliberate reflection on learning about the content knowledge of the cases. In contrast to our results, they found that practicing with deliberate reflection increased diagnostic accuracy on cases diagnosed one week later. In that study the future test cases were similar to the practice cases, whereas in our study we also included unrelated test cases. Therefore, it can be that the benefit of deliberate reflection on improving future diagnostic accuracy is only due to learning about the specific content of the cases rather than the reflective procedure and does not transfer to cases with unrelated diseases. However, Ibiapina et al. did not test the effect on unrelated cases and we do not know whether it also had an effect on the students’ reasoning process. Future studies should conduct the present study with students, to see whether practicing with deliberate reflection is effective in teaching reflective reasoning if participants are less experienced than residents are.

To sum up, the results of the present study showed that for residents in the general practice training, practicing with deliberate reflection by explaining it on video did not increase reflective reasoning on future cases. It could be that the residents did not yet adopt the procedure and that more practice is needed, or that the residents did not feel the need to apply the procedure in the test phase. Another explanation is that the control condition also engaged in reflective reasoning during the test phase, and that the added benefit of deliberate reflection is too small to find an effect.

### Electronic supplementary material

Below is the link to the electronic supplementary material.

Supplementary file1 (DOC 19 kb)

## Appendix

See Fig. 1, Tables 1 and 2.
