# Supplementary material for: Learning deliberate reflection in medical diagnosis: does learning-by-teaching help?
Source: Adv Health Sci Educ Theory Pract. 2022 Aug 1;28(1):13–26. doi: 10.1007/s10459-022-10138-2 (PMC9992049; doi:10.1007/s10459-022-10138-2)
Supplement: Supplementary file 1 — Supplementary file1 (DOC 19 kb) [file 10459_2022_10138_MOESM1_ESM.doc]

Supplementary material

Supplementary Table 1
Diagnoses and symptoms shown during the prior knowledge assessment.

| Correct diagnoses and chief complaints that appear in the cases | Other diagnoses and symptoms (filler) |
| --- | --- |
| Diarrhoea | Constipation |
| Irritable bowel syndrome (IBS) | Abdominal pain |
| Inflammatory bowel disease (IBD) | Gastroenteritis |
| Chronic pancreatitis | Infection of the upper respiratory tract |
| Shortness of breath | Chronic Obstructive Pulmonary Disease |
| Infection of the lower respiratory tract | Anaemia |
| Pulmonary embolism | Pain on the chest |
| Rash in the face | Depression |
| Rosacea | Eating disorder |
| Tremor in hand | Vaginal complaints |
| Multiple sclerosis | Vaginal fungal infection |
| Facial paralysis | Sexually Transmitted Diseases |
| Idiopathic peripheral facial paralysis (IPAV) | Erectile dysfunction |
|  | Headache |
|  | Dizziness |
|  | Thumb base instability |
|  | Shingles |
|  | Acne |
|  | Pain in thumb |
|  | Weak muscles |
|  | Hyperthyroidism |
|  | Cerebrovascular Accident (CVA) |
|  | Lyme disease |
|  | Diverticulitis |
|  | Asthma |
|  | Heart failure |
|  | Palpitations |
|  | Anxiety / panic disorder |
|  | Vaginal discharge |
|  | Amenorrhea |
|  | Bacterial vaginosis |
|  | Benign Paroxysmal Position Vertigo (BPPD) |
|  | Pregnancy |
|  | Skin rash |
|  | Scarlet fever |
|  | Lower back pain |
|  | Pain in legs |
|  | Spondylodiscitis |
|  | Spinal canal stenosis / neurogenic claudication |
|  | Turn dizziness |

Supplementary Table 2 *Overview of the medical conditions of the cases used during the different session. Chief complaints are given in parenthesis. Three cases in the learning phase have only been shown to the control condition and are market with an asterisk.*

| Learning session | Post-test session |
| --- | --- |
| Chronic pancreatitis  (diarrhoea) | Chronic pancreatitis  (diarrhoea) |
| Inflammatory bowel disease  (diarrhoea) | Inflammatory bowel disease  (diarrhoea) |
| Irritable bowel syndrome  (diarrhoea) | Infection of the lower respiratory tract  (shortness of breath) |
| Bell's palsy*  (facial paralysis) | Pulmonary embolism  (shortness of breath) |
| Rosacea* (Rash in the face) |  |
| Multiple sclerosis*  (tremor in hand) |  |

*Note. Each case was different, i.e. described a different patient.*

| You haven't seen Mrs. Alkema (27 years old) in a while. Since the last time you spoke with her, she completed her law studies and is working long days at a law firm since. She enjoys her work. She likes to really delve into a case and get the most out of it. Recently she moved in a new house in a village together with her girlfriend, and every morning she gets into the car to join the traffic jam towards the city. But this morning she has an appointment at your practice and she seems slightly irritated that your schedule is 10 minutes delayed.  She quickly explains why she came: severe abdominal pain. But really severe; so bad that at these moments she just has to go to the bathroom. Even when she is in a meeting. Once she even had to leave the courtroom, for which she feels embarrassed. However, it always brings her some relief for a moment. At these times, she has somewhat thin stool. Occasionally there is some blood on it. You ask where the abdominal pain is located; that is clear, around the navel.  "This can’t go on; can’t you just refer me to a specialist?" When you ask her, it appears that she has been suffering from this for more than half a year. To keep going, she has used Naproxen 250mg twice a day for the last 2 weeks. Her menstruation is not as much as it used to be, sometimes it skips. To lose weight, she has been following a diet for the last 2 months, which you have not heard of before. It is working; she has lost some weight. At the end of the consultation, you remember that her mother also often had abdominal pain.  **Physical examination:**  During the physical examination, you find pressure pain near the descending colon, but no further abnormalities. The checks are good.  **Additional tests:**  You decide to order laboratory tests, in the hope that the results will be reassuring. You do not have the results yet. |
| --- |

*Supplementary Figure 1.* Translation of a case of irritable bowel syndrome, with as chief complaint diarrhoea. This case has been shown during the learning session.

| Diverticulitis (complicated/ uncom-plicated) | Irritable bowel syndrome (IBS) | Inflammatory bowel disease (IBD) | Diagnosis |
| --- | --- | --- | --- |
| - Pressure pain in left lower abdomen - Recurrent course - No alarm symptoms - Rectal blood loss | - Duration and frequency of abdominal pain and diarrhoea - Complaints reduce after defecation - Complaints always come with diarrhoea - Age <50 years - Hard working (stress) - Abdominal pain around navel - Pressure pain colon descendens - Mother too had stomach ache | - Diarrhoea with intervals - Rectal blood loss | Which findings from the case speak **for** this diagnosis? |
|  | - Rectal blood loss (but probably hemorrhoid) | - Complaints always temporary - No involuntary weight loss or other additional complaints | Which findings from the case speak **against** this diagnosis? |
| - More and sharper pain - More pressure and release pain - Fever - Abdominal resistance | - Mucus with stool - Flatulence - Depending on food / stress | - Alarm signals (weight loss, tired) - Perianal abnormality - Fever - Abdominal resistance - Family members with IBD | Which further findings would you expect if your diagnosis was correct, which are **missing** for this patient? |
|  |  |  | Finally: Order of likelihood (1 = most likely) |

Supplementary Figure 2. Translation of the worked-out deliberate reflection example shown to participants in the learning-by-teaching condition during the learning session.
